# The bloody mess of red blood cell transfusion

**DOI:** 10.1186/s13054-017-1912-x

**Published:** 2017-12-28

**Authors:** Susilo Chandra, Hrishikesh Kulkarni, Martin Westphal

**Affiliations:** 1 Department of Anesthesiology and Intensive Care, Cipto Mangunkusumo General Hospital, University of Indonesia, Medical Faculty, Jakarta, Indonesia; 2 Fresenius Kabi, Bad Homburg, Germany; 3 Department of Anesthesiology, Intensive Care and Pain Medicine, University of Muenster, Muenster, Germany

## Abstract

Red blood cell (RBC) transfusion might be life-saving in settings with acute blood loss, especially uncontrolled haemorrhagic shock. However, there appears to be a catch-22 situation reflected by the facts that preoperative anaemia represents an independent risk factor for postoperative morbidity and mortality, and that RBC transfusion might also contribute to adverse clinical outcomes. This dilemma is further complicated by the difficulty to define the “best” transfusion trigger and strategy. Since one size does obviously not fit all, a personalised approach is merited. Attempts should thus be made to critically reflect on the pros and cons of RBC transfusion in each individual patient. Patient blood management concepts including preoperative, intraoperative and postoperative optimisation strategies involving the intensive care unit are warranted and are likely to provide benefits for the patients and the healthcare system. In this context, it is important to consider that “simply” increasing the haemoglobin content, and in proportion oxygen delivery, may not necessarily contribute to a better outcome but potentially the contrary in the long term. The difficulty lies in identification of the patients who might eventually profit from RBC transfusion and to determine in whom a transfusion might be withheld without inducing harm. More robust clinical data providing long-term outcome data are needed to better understand in which patients RBC transfusion might be life-saving vs life-limiting.

Red is the color in which the interior of the body is painted. If an operation be thought of as a painting in progress, and blood red the color on the brush, it must be suitably restrained and attract no undue attention; yet any insufficiency of it will increase the perishability of the canvas.

Richard Selzer, *Letters to a Young Doctor*

## Background

Red blood cell (RBC) transfusion is one of the most iconic intravenous treatments in medicine. In life-threatening conditions of massive blood loss, state-of-the-art RBC transfusion might be the most prominent option to prevent immediate death. Based on this tradition, a healthcare provider treating an anaemic surgical or critically ill patient may consider RBC transfusion as a life-saving treatment. Increasing evidence, however, indicates that RBC transfusion might also induce harm in a dose-dependent manner [1–4]. Therefore, any transfusion decision presupposes not only a thorough consideration of pros and cons, but also a careful assessment of potential alternatives.

## Anaemia prevalence in the general population and surgical patients

The World Health Organization defines anaemia as a decrease of haemoglobin (Hb) concentration below 13 g/dl in men and below 12 g/dl in women and children older than 6 years. [5] Anaemia in the general population is typically caused by chronic bleeding, several infectious and chronic diseases that depress haematopoiesis like chronic kidney disease as well as genetic disorders. In the clinical setting, acute anaemia usually results from severe bleeding and/or dilutional effects secondary to fluid overload. Iron deficiency, however, is the main cause for anaemia and responsible for about 50% of all cases globally [6]. Iron deficiency itself is a complex condition that may result from an absolute lack of iron, or from functional iron deficiencies like reduced gastrointestinal uptake, insufficient transport to the sites of erythropoiesis or incomplete incorporation into Hb [7, 8].

**Fig. 1:**
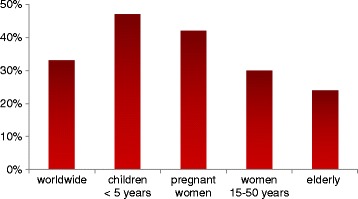
Prevalence of anaemia. Prevalence of anaemia worldwide and in patients at high risk. Figure based on [6]

It is important to know that iron deficiency may result from several common conditions like inadequate dietary uptake (e.g. vegetarianism/veganism) [9], increased losses (e.g. menorrhagia) [10], impaired iron absorption, as evidenced in inflammatory bowel disease [11], or increased need, as during pregnancy [9, 12]. The worldwide prevalence of anaemia is 33%, with the highest occurrence in children younger than 5 years (prevalence 47%) and pregnant women (prevalence 42%). Other groups at high risk of anaemia are non-pregnant women aged 15–50 (prevalence 30%) or the elderly (prevalence 24%) (Fig. 1) [6].

Because anaemia is common in the general population, it is also highly relevant in hospitalised patients. In this context, a prospective observational trial in elderly patients found that even mild anaemia (Hb 10–12 g/dl in females and 10–13 g/dl in males) was associated with a significantly higher risk of hospitalisation (hazard ratio 1.44) and even a significant increase in mortality [13]. Similar results have been found in a retrospective study of 227,425 patients undergoing non-cardiac surgery [14]. In this study, anaemia was independently associated with increased morbidity and mortality. Notably, a meta-analysis including almost 1 million surgical patients confirmed these findings. In the latter analysis, anaemia was present in 39% of surgical patients and was associated with increased mortality (odds ratio (OR) 2.9), acute kidney injury (OR 3.75) and infections (OR 1.93) [15].

In view of the published literature, anaemia is common in the general population and relevant for patient outcome. In the hospital, acute anaemia is often treated by RBC transfusions, because it has been proven to increase Hb levels in a timely manner. This therapeutic approach, however, usually does not address the underlying disease that triggered anaemia and may carry its own risks that have to be taken into account.

## Transfusion in surgery

In the setting of acute uncontrollable haemorrhage, RBC transfusion frequently saves lives [16]. This common understanding is based on strong clinical experience, albeit on weak study evidence, because denying transfusions in a life-threatening condition for the sake of a trial would be undoubtedly unethical. A hypothetical experiment based on a national survey in the Netherlands suggests that the risk of maternal death in obstetric care would increase 6.5-fold if no RBC transfusions were available [17]. Physiological clinical data also support this line of thought. In this regard, an observational study demonstrated improved systemic oxygen-carrying capacity and microcirculation after RBC transfusion in cardiac surgery [18].

However, improved microcirculation is just one effect of RBC transfusions and might be offset by more subtle sequels like renal dysfunction [19]. In fact, data derived from general surgical populations suggest significant risks associated with RBC transfusion [20]. A retrospective analysis of 10,100 surgical patients with severe anaemia (packed cell volume/haematocrit < 30%) found an increased risk of death associated with intraoperative transfusions (OR 1.29) as well as more postoperative complications [20]. Concordant results have been reported in patients undergoing cardiac surgery [21], and resulted in recommendations to restrict transfusions in haemodynamically stable patients [22].

The evidence generated from these observational studies is further supported by the identification of a dose-dependent detrimental effect of RBC transfusion in patients undergoing coronary artery graft surgery [3]. Notably, there was no increased risk of death for low to moderate infusion of blood products (≤6 units), but increased mortality following higher amounts of RBC transfusion. Similarly, a recent study has linked RBC transfusion to pneumonia in a dose-dependent manner, thus indicating that transfusions may impair the immune system and/or damage the lung by fluid overload [4].

The (potentially deleterious) effects of RBC transfusion may depend on the patient risk profile per se. It seems plausible that “one size does not fit all” and that certain patients might benefit from a transfusion whereas others may be harmed. Therefore, identification of the patients profiting from RBC transfusion—and vice versa—seems to be crucial. Some guidance is provided by a sub-study of the Transfusion Requirements After Cardiac Surgery (TRACS) study [2]. When patient outcomes were analysed separately by age (younger or older than 60 years) and by liberal (Hb 10 g/dl) versus restrictive (Hb 8 g/dl) transfusion goals, the authors found no significant difference for the primary outcome of 30-day mortality combined with severe morbidity. However, they reported a higher incidence of cardiogenic shock in older patients with a lower Hb target. This finding is supported by the recent meta-analysis of Docherty et al. [1]. While the pooled risk ratio in this analysis for the association between transfusion thresholds and 30-day mortality increased non-significantly (1.15; 95% confidence interval 0.88 to 1.50, *p* = 0.50), the risk of acute coronary syndrome in patients managed with restrictive transfusion threshold increased significantly (risk ratio 1.78; 95% confidence interval 1.18 to 2.70, *p* = 0.01). The authors concluded that a restrictive transfusion regimen with an Hb threshold below 8 g/dl may not be safe in patients with acute coronary syndrome or chronic cardiovascular disease.

Thus, despite controversial reports in the literature, it might well be that elderly patients with cardiovascular co-morbidities may profit from a more liberal transfusion regimen, while other surgical patients do not.

## Transfusion in critically ill patients

The landmark study of RBC transfusions in critically ill patients was published by Hébert et al. [23] in 1999. The study demonstrated better survival with a restrictive transfusion strategy (Hb 7–9 g/dl) compared to a liberal transfusion strategy (Hb 10–12 g/dl). Patients included in this study had no substantial blood loss (as indicated by a drop in Hb of more than 3 g/dl or need for 3 units of RBCs or more within 12 hours before enrolment). However, this study was conducted when leucocyte depletion was not practised routinely.

**Fig. 2:**
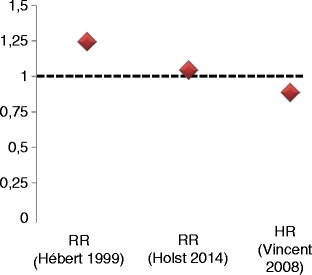
Risk of death for liberal vs restrictive transfusion in critically ill patients. Relative risk of death for patients with a liberal transfusion regime compared to a restrictive transfusion regime (23.3% vs 17.7%, RR 1.25) [23] (Hébert 1999). Relative risk of death for patients with a liberal transfusion regimen (higher Hb threshold, 45%) compared to a restrictive infusion strategy (lower Hb threshold, 43%) (RR 1.05) [25] (Holst 2014). Hazard ratio for 30-day survival 0.89 [24] (Vincent 2008). HR hazard ratio, RR relative risk. Figure based on [23–25]

Approximately 10 years later, an analysis of the observational Sepsis Occurrence in Acutely Ill Patients (SOAP) study suggested a different conclusion for critically ill patients [24]. The raw data showed a higher intensive care unit and hospital mortality for patients receiving RBCs. After multivariate analysis, however, a higher 30-day survival was found for those patients receiving RBCs during their ICU stay. The authors hypothesised that changes in preparation of RBCs, namely better reduction of viral load and leucodepletion, may be the cause for their findings that contrasted with the results of the Hébert et al. study (Fig. 2).

In 2014, the Transfusion Requirements in Septic Shock (TRISS) trial rigorously tested the actual effect of transfusions on outcome in critically ill patients [25]. Patients with septic shock were randomly assigned to receive transfusions based either on a low threshold algorithm (Hb < 7 g/dl) or a high threshold algorithm (Hb < 9 g/dl). Thus, both groups were treated within the range of the restrictive strategy used in the Hébert et al. study. This approach resulted in a median transfusion of 1 RBC unit for the low threshold group and 4 RBC units for the high threshold group. The primary endpoint (i.e. 90-day mortality) was reached in 43% of the patients with lower threshold compared to 45% with the higher threshold (*p* = 0.44). Interestingly, the investigators also found no difference in the number of adverse or ischaemic events. These results were consistent with those of the pre-specified subgroups with and without chronic cardiovascular disease, 70 years of age or younger and patients with a Simplified Acute Physiology Score (SAPS) II score below or above 53 at baseline. These results suggest that RBC transfusion might have been safe in the studied patients. Conversely, a recent retrospective analysis indicates that RBC transfusions may be an independent risk factor in critically ill children [26].

Based on the vast majority of the published literature, restrictive transfusions seem to be adequate for critically ill patients [27, 28]. Increasing the dose of RBCs in the absence of uncontrollable blood loss does not, at least, appear to improve outcome.

## Management of anaemia to reduce RBC transfusions

Transfusion of RBCs is still a common and effective measure to treat acute anaemia. However, evidence suggests that RBC transfusion in stable patients may increase morbidity and long-term mortality in a dose-dependent manner [1–3]. Thus, it should be aimed at avoiding transfusion in stable (elective surgery) patients whenever possible. Such a strategy should be based on three guiding principles: first, the detection and treatment of preoperative anaemia; second, the reduction of perioperative blood loss and blood loss due to diagnostic procedures in the ICU; and third, the leverage of patient-specific physiological reserves to improve oxygenation [29].

Before elective procedures, pre-existing anaemia should be identified and treated with nutritional interventions, iron or erythropoietin as appropriate [30, 31]. However, it needs to be taken into account that erythropoietin may impair disease control in patients with malignancies and is, therefore, contraindicated [30, 32]. Concomitant medications, family history and co-morbidities should always be checked carefully to allow a better identification of the risk for increased blood loss in surgical patients. Whenever possible, the patients’ physiologic reserve (e.g. pulmonary and cardiac function) should be optimised. Minimisation or even avoidance of (allogeneic) transfusions might be achieved by ideal preparation of the patient. Such strategies should be based on the expected probability of transfusion. In patients at high risk of transfusion (≥ 10%), a careful evaluation of the patient’s condition is mandatory 4–8 weeks prior to the procedure to allow sufficient time for corrective measures [28, 30, 31].

Preventing, or at least limiting, intraoperative blood loss is crucial whenever possible. In this regard, minimally invasive surgical techniques [33, 34], the use of haemostatic agents such as tranexamic acid [35] and warming systems to prevent a drop in core body temperature are useful measures to effectively reduce blood loss [36–38]. In addition, cell salvage and reinfusion techniques may further reduce the need for allogeneic RBC transfusion in patients undergoing major surgery [28].

In the postoperative phase, an adequate oxygen-carrying capacity of the patient should be targeted [28]. In parallel, measures initiated preoperatively like optimised iron supply may have to be continued. All physiological approaches relevant for surgical patients are also to be considered for critically ill patients. Furthermore, restrictive transfusion triggers might be applied [28] and tranexaemic acid used to limit blood loss [27].

**Fig. 3:**
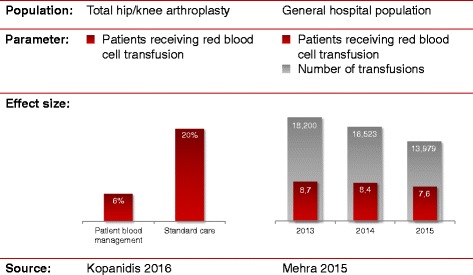
Effect sizes of recent patient blood management programmes. Recent publications on the effects of multimodal PBM programmes have demonstrated substantial reductions in the number of patients receiving RBC transfusions in an RCT and in the number of transfusions utilised at a hospital. Based on [39] (Kopanidis 2016) and [40] (Mehra 2015)

The beneficial effects of standardised patient blood management (PBM) have been demonstrated in a recent study in 200 patients undergoing hip and knee arthroplasty [39]. In this context, it has been reported that a standardised algorithm for the management of anaemia prevented transfusions in significantly more patients (6 vs 20%, *p* = 0.003). Although patients managed by this algorithm received fewer transfusions, the postoperative and discharge Hb values were higher. In another multicentre cohort study including more than 100,000 patients, the implementation of a PBM system reduced allogeneic blood transfusions significantly by 27% and resulted in significant cost savings [40] (Fig. 3).

## Conclusion

Anaemia is a common phenomenon in the general population and represents a relevant risk factor for increased morbidity and mortality, especially in patients undergoing major surgery [6, 13–15]. The fact that RBC transfusions may likewise impair outcome in a dose-dependent manner results in a dilemma [1–4]. Therefore, clinical strategies should aim at better identifying patients who are likely to profit from RBC transfusion. At the same time, it is of utmost importance to identify patients in whom transfusion might be futile and especially those likely to be harmed by unnecessary transfusions. Until conclusive evidence is available, the concept of “less may be best” or “as much as needed and as little as possible” should be applied. Since there is no doubt that our own blood is still the best fluid to have in our veins [41], clinical strategies, such as PBM concepts, should aim at keeping it there. This “medical art” might solve the bloody mess of RBC transfusion.

## Abbreviations

Hb: Haemoglobin
OR: Odds ratio
PBM: Patient blood management
RBC: Red blood cell
SOAP: Sepsis Occurrence in Acutely Ill Patients
TRACS: Transfusion Requirements After Cardiac Surgery
TRISS: Transfusion Requirements in Septic Shock
WHO: World Health Organization

## References

[1] Docherty AB, O’Donnell R, Brunskill S, Trivella M, Doree C, Holst L, Parker M, Gregersen M, Pinheiro de Almeida J, Walsh TS, Stanworth SJ (2016). Effect of restrictive versus liberal transfusion strategies on outcomes in patients with cardiovascular disease in a non-cardiac surgery setting: systematic review and meta-analysis. BMJ.

[2] Nakamura RE, Vincent J, Fukushima JT, de Almeida JP, Franco RA, Lee Park C, Osawa EA, Pinto Silva CM, Costa Auler JO, Landoni G, Barbosa Gomes Galas FR, Filho RK, Hajjar LA (2015). A liberal strategy of red blood cell transfusion reduces cardiogenic shock in elderly patients undergoing cardiac surgery. J Thorac Cardiovasc Surg.

[3] Weightman WM, Gibbs NM, Sheminant MR, Newman MA, Grey DE (2009). Moderate exposure to allogeneic blood products is not associated with reduced long-term survival after surgery for coronary artery disease. Anesthesiology.

[4] Likosky DS, Paone G, Zhang M, Rogers AM, Harrington SD, Theurer PF, DeLucia A, Fishstrom A, Camaj A, Prager RL (2015). Red blood cell transfusions impact pneumonia rates after coronary artery bypass grafting. Ann Thorac Surg.

[5] World Health Organization (WHO). Haemoglobin concentrations for the diagnosis of anaemia and assessment of severity. http://www.who.int/vmnis/indicators/haemoglobin.pdf.

[6] Kassebaum NJ, Jasrasaria R, Naghavi M, Wulf SK, Johns N, Lozano R, Regan M, Weatherall D, Chou DP, Eisele TP, Flaxman SR, Pullan RL, Brooker SJ, Murray CJ (2014). A systematic analysis of global anemia burden from 1990 to 2010. Blood.

[7] Shander A, Knight K, Thurer R, Adamson J, Spence R (2004). Prevalence and outcomes of anemia in surgery: a systematic review of the literature. Am J Med.

[8] Clevenger B, Mallett SV, Klein AA, Richards T (2015). Patient blood management to reduce surgical risk. Br J Surg.

[9] Zimmermann MB, Hurrell RF (2007). Nutritional iron deficiency. Lancet (London, England).

[10] Marret H, Fauconnier A, Chabbert-Buffet N, Cravello L, Golfier F, Gondry J, Agostini A, Bazot M, Brailly-Tabard S, Brun J, de Raucourt E, Gervaise A, Gompel A, Graesslin O, Huchon C, Lucot J, Plu-Bureau G, Roman H, Fernandez H (2010). Clinical practice guidelines on menorrhagia: management of abnormal uterine bleeding before menopause. Eur J Obstet Gynecol Reprod Biol.

[11] Crichton RR, Danielson BG, Geisser P (2008). Iron therapy: with special emphasis on intravenous administration (UNI-MED Science).

[12] Milman N (2006). Iron and pregnancy—a delicate balance. Ann Hematol.

[13] Riva E, Tettamanti M, Mosconi P, Apolone G, Gandini F, Nobili A, Tallone MV, Detoma P, Giacomin A, Clerico M, Tempia P, Guala A, Fasolo G, Lucca U (2009). Association of mild anemia with hospitalization and mortality in the elderly: the Health and Anemia population-based study. Haematologica.

[14] Musallam KM, Tamim HM, Richards T, Spahn DR, Rosendaal FR, Habbal A, Khreiss M, Dahdaleh FS, Khavandi K, Sfeir PM, Soweid A, Hoballah JJ, Taher AT, Jamali FR (2011). Preoperative anaemia and postoperative outcomes in non-cardiac surgery: a retrospective cohort study. Lancet (London, England).

[15] Fowler AJ, Ahmad T, Phull MK, Allard S, Gillies MA, Pearse RM (2015). Meta-analysis of the association between preoperative anaemia and mortality after surgery. Br J Surg.

[16] Vymazal T (2015). Massive hemorrhage management—a best evidence topic report. Ther Clin Risk Manag.

[17] Hendriks J, Zwart JJ, Briët E, Brand A, van Roosmalen J (2013). The clinical benefit of blood transfusion: a hypothetical experiment based on a nationwide survey of severe maternal morbidity. Vox Sang.

[18] Yuruk K, Almac E, Bezemer R, Goedhart P, de Mol B, Ince C (2011). Blood transfusions recruit the microcirculation during cardiac surgery. Transfusion.

[19] Thongprayoon C, Cheungpasitporn W, Gillaspie EA, Greason KL, Kashani KB (2016). Association of blood transfusion with acute kidney injury after transcatheter aortic valve replacement: a meta-analysis. World J Nephrol.

[20] Glance LG, Dick AW, Mukamel DB, Fleming FJ, Zollo RA, Wissler R, Salloum R, Meredith UW, Osler TM (2011). Association between intraoperative blood transfusion and mortality and morbidity in patients undergoing noncardiac surgery. Anesthesiology.

[21] Murphy GJ, Reeves BC, Rogers CA, Rizvi SI, Culliford L, Angelini GD (2007). Increased mortality, postoperative morbidity, and cost after red blood cell transfusion in patients having cardiac surgery. Circulation.

[22] Rawn JD (2007). Blood transfusion in cardiac surgery. Circulation.

[23] Hébert PC, Wells G, Blajchman MA, Marshall J, Martin C, Pagliarello G, Tweeddale M, Schweitzer I, Yetisir E (1999). A multicenter, randomized, controlled clinical trial of transfusion requirements in critical care. Transfusion Requirements in Critical Care Investigators, Canadian Critical Care Trials Group. N Engl J Med.

[24] Vincent J, Sakr Y, Sprung C, Harboe S, Damas P (2008). Are blood transfusions associated with greater mortality rates? Results of the Sepsis Occurrence in Acutely Ill Patients study. Anesthesiology.

[25] Holst LB, Haase N, Wetterslev J, Wernerman J, Guttormsen AB, Karlsson S, Johansson PI, Aneman A, Vang ML, Winding R, Nebrich L, Nibro HL, Rasmussen BS, Lauridsen JR, Nielsen JS, Oldner A, Pettilä V, Cronhjort MB, Andersen LH, Pedersen UG, Reiter N, Wiis J, White JO, Russell L, Thornberg KJ, Hjortrup PB, Müller RG, Møller MH, Steensen M, Tjäder I (2014). Lower versus higher hemoglobin threshold for transfusion in septic shock. N Engl J Med.

[26] Rajasekaran S, Kort E, Hackbarth R, Davis AT, Sanfilippo D, Fitzgerald R, Zuiderveen S, Ndika AN, Beauchamp H, Olivero A, Hassan N (2016). Red cell transfusions as an independent risk for mortality in critically ill children. J Intensive Care.

[27] National Blood Authority. Patient Blood Management Module 4 Critical Care. https://www.blood.gov.au/pbm-module-4.

[28] Kozek-Langenecker SA, Afshari A, Albaladejo P, Santullano CAA, de Robertis E, Filipescu DC, Fries D, Görlinger K, Haas T, Imberger G, Jacob M, Lancé M, Llau J, Mallett S, Meier J, Rahe-Meyer N, Samama CM, Smith A, Solomon C, Van Der Linden P, Wikkelsø AJ, Wouters P, Wyffels P (2013). Management of severe perioperative bleeding: guidelines from the European Society of Anaesthesiology. Eur J Anaesthesiol.

[29] Meybohm P, Richards T, Isbister J, Hofmann A, Shander A, Goodnough LT, Muñoz M, Gombotz H, Weber CF, Choorapoikayil S, Spahn DR, Zacharowski K (2017). Patient blood management bundles to facilitate implementation. Transfus Med Rev.

[30] Hoppe JD, Scriba PC, Klueter H. Querschnitts-Leitlinien zur Therapie mit Blutkomponenten und Plasmaderivaten: Mit 19 Tabellen. 4th ed. Köln: Dt. Ärzte-Verl; 2009.

[31] Goodnough LT, Maniatis A, Earnshaw P, Benoni G, Beris P, Bisbe E, Fergusson DA, Gombotz H, Habler O, Monk TG, Ozier Y, Slappendel R, Szpalski M (2011). Detection, evaluation, and management of preoperative anaemia in the elective orthopaedic surgical patient: NATA guidelines. Br J Anaesth.

[32] Henke M, Laszig R, Rübe C, Schäfer U, Haase K, Schilcher B, Mose S, Beer KT, Burger U, Dougherty C, Frommhold H (2003). Erythropoietin to treat head and neck cancer patients with anaemia undergoing radiotherapy: randomised, double-blind, placebo-controlled trial. Lancet (London, England).

[33] Meybohm P, Herrmann E, Steinbicker AU, Wittmann M, Gruenewald M, Fischer D, Baumgarten G, Renner J, Van Aken HK, Weber CF, Mueller MM, Geisen C, Rey J, Bon D, Hintereder G, Choorapoikayil S, Oldenburg J, Brockmann C, Geissler RG, Seifried E, Zacharowski K (2016). Patient blood management is associated with a substantial reduction of red blood cell utilization and safe for patient’s outcome: a prospective, multicenter cohort study with a noninferiority design. Ann Surg.

[34] Shander A, Javidroozi M, Perelman S, Puzio T, Lobel G (2012). From bloodless surgery to patient blood management. Mt Sinai J Med.

[35] Henry DA, Carless PA, Moxey AJ, O’Connell D, Stokes BJ, Fergusson DA, Ker K (2011). Anti-fibrinolytic use for minimising perioperative allogeneic blood transfusion. Cochrane Database Syst Rev.

[36] Rajagopalan S, Mascha E, Na J, Sessler DI (2008). The effects of mild perioperative hypothermia on blood loss and transfusion requirement. Anesthesiology.

[37] Cavallini M, Baruffaldi Preis FW, Casati A (2005). Effects of mild hypothermia on blood coagulation in patients undergoing elective plastic surgery. Plast Reconstr Surg.

[38] Winkler M, Akça O, Birkenberg B, Hetz H, Scheck T, Arkiliç CF, Kabon B, Marker E, Grübl A, Czepan R, Greher M, Goll V, Gottsauner-Wolf F, Kurz A, Sessler DI (2000). Aggressive warming reduces blood loss during hip arthroplasty. Anesth Analg.

[39] Kopanidis P, Hardidge A, McNicol L, Tay S, McCall P, Weinberg L (2016). Perioperative blood management programme reduces the use of allogenic blood transfusion in patients undergoing total hip and knee arthroplasty. J Orthop Surg Res.

[40] Mehra T, Seifert B, Bravo-Reiter S, Wanner G, Dutkowski P, Holubec T, Moos RM, Volbracht J, Manz MG, Spahn DR (2015). Implementation of a patient blood management monitoring and feedback program significantly reduces transfusions and costs. Transfusion.

[41] Frenzel T, Van Aken H, Westphal M (2008). Our own blood is still the best thing to have in our veins. Curr Opin Anaesthesiol.

